# Targeted Therapy in Hematological Malignancies: From Basic Research to Clinical Practice

**DOI:** 10.1155/2015/157570

**Published:** 2015-09-08

**Authors:** Haiqing Ma, Saradhi Mallampati, Gang An, Jin Wang

**Affiliations:** ^1^Department of Oncology, The Fifth Affiliated Hospital of Sun Yat-sen University, Zhuhai, China; ^2^Department of Laboratory Medicine, The University of Texas MD Anderson Cancer Center, Houston, TX, USA; ^3^Institute of Hematology & Blood Diseases Hospital, Chinese Academy of Medical Science & Peking Union Medical College, Tianjin, China; ^4^Department of Translational Molecular Pathology, The University of Texas MD Anderson Cancer Center, Houston, TX, USA

Targeted therapy in hematological malignancies has been a forerunner and still remains in the forefront of ongoing research. Over the past decades, many advances in basic research have boosted the advancement of targeted therapy in clinical studies. To date, targeted therapy has provided benefits for patients with hematological malignancies either as the first-line treatment or in combination with chemotherapy. Undoubtedly, in the near future, customized targeted therapy will play a more important role in the treatment of hematological malignancies.

This special issue on targeted therapy in hematological malignancies includes reviews and original research articles that describe novel molecular targets, innovative technologies, recent clinical trials, mechanisms of drug resistance, and other advances in targeted therapy for hematological malignancies.

Dr. J.-F. Rossi in the review article entitled “Targeted Therapies in Adult B-Cell Malignancies” summarizes currently targeted molecules in adult B-cell malignancies and didactically describes the various cell compartments (membrane versus cytosol) that can be targeted and explains how most of the molecular pathways either proximal or distal to B-cell receptor (BCR) can be blocked with targeted therapies. The review also includes a highly informative synopsis of all the relevant clinical trials and will be extremely useful to all the readers, especially to those in the field of hematology oncology, both in the clinic and in research.

Dr. O. Annibali et al. report the outcome of using Rituximab as a first-line systemic treatment in a series of mucosa-associated lymphoid tissue-type ocular adnexal lymphomas (MALT OALs) with additional maintenance. OALs are rare types of lymphoma, for which the specific treatment options were not currently available. Only few cases were reported previously on the efficacy of Rituximab immunotherapy as a single-agent in primary localized MALT OALs. The response duration in the previously reported trials was short which could have been due to the absence of additional maintenance in those studies. This study clearly indicates that the maintenance therapy with Rituximab ensures prolonged remission.

Dr. D. Tusé et al. report the evaluation of novel plant-based conjugate vaccines for targeted treatment of B-cell follicular lymphoma (FL) in a phase I safety and immunogenicity clinical study. This phase I study was exceedingly successful as none of the patients suffered any serious adverse events related to vaccination. The customized idiotype vaccines produced by means of the magnICON, a plant-based expression technology, are very promising for they are readily and economically manufactured, safe, well tolerated, and immunogenic.

Dr. Z.-X. Yan et al. report that overexpression of miR181 in human T-cell leukemia/lymphoma is related to increased AKT phosphorylation. Malignant T cells overexpressing miR181 exhibited multiple chemoresistance mechanisms through modulation of AKT activity. Moreover, in isogenic doxorubicin-resistant cell lines developed, the relative resistance to doxorubicin and other chemotherapeutic agents was associated with increased miR181 expression and subsequent AKT activation. So miR181 could serve as a useful biomarker and a potential therapeutic target in treating T-cell malignancies resistant to chemotherapy.

Dr. S. Wu et al. demonstrate that subcutaneous administration of bortezomib is not inferior to its intravenous administration and confirmed that bortezomib and thalidomide plus dexamethasone regimen is highly active and well tolerated as induction therapy in patients with multiple myeloma.

With all these novel and well-done research and clinical trials, this issue promises to be an enlightening read for clinicians and scientists.

